# Pyogenic spondylitis caused by *Klebsiella pneumoniae*: should the possibility of hypervirulent *Klebsiella pneumoniae* be considered?

**DOI:** 10.1186/s12879-022-07785-6

**Published:** 2022-10-27

**Authors:** Joo-Hee Hwang, Seung Yeob Lee, Jaehyeon Lee, Jeong-Hwan Hwang

**Affiliations:** 1 Department of Internal Medicine, Jeonbuk National University Medical School and Hospital, 20 Geonjiro, Deokjin-gu, 54907 Jeonju-si, Jeollabuk-do South Korea; 2 Research Institute of Clinical Medicine, Biomedical Research Institute of Jeonbuk National University Hospital, 20 Geonjiro, Deokjin-gu, 54907 Jeonju-si, Jeollabuk-do South Korea; 3 Department of Laboratory Medicine, Jeonbuk National University Medical School and Hospital, 20 Geonjiro, Deokjin-gu, 54907 Jeonju-si, Jeollabuk-do South Korea

**Keywords:** Pyogenic spondylitis, *Klebsiella pneumoniae*, Hypervirulence

## Abstract

**Background:**

*Klebsiella pneumoniae* is rare but the second most common causative agent among gram-negative bacteria that cause pyogenic spondylitis. However, there are no available studies on the serotype, virulence factors, and clinical characteristics associated with *K. pneumoniae*-caused pyogenic spondylitis. Accordingly, we investigated the clinical characteristics of pyogenic spondylitis, K1 and K2 serotypes, and virulence factors of *K. pneumoniae*.

**Methods:**

We reviewed the microbiological reports of specimens collected between January 2014 and December 2019 as well as the medical records of patients with pyogenic spondylitis caused by *K. pneumoniae*. We also evaluated K1 and K2 serotypes and the virulent genes *rmpA*, *iutA*, *mrkD*, *ybtS*, *entB*, and *kfu*. Strains that possessed *rmpA* and *iutA* were defined as hypervirulent *K. pneumoniae*.

**Results:**

Six patients with pyogenic spondylitis caused by *K. pneumoniae* were enrolled in the study. The capsular serotypes K1 and K2 were present in 66.7% (4/6) of cases, and the hypervirulent strains were present in 88.3% (5/6) of cases. All patients had community-acquired infections, and all strains isolated were susceptible to antimicrobial agents. Intravenous antibiotic treatment continued for 2–7 weeks, and no patient underwent decompressive operation or surgical debridement. There was no recurrence. One patient died from pneumonia with a septic lung.

**Conclusion:**

Hypervirulent *K. pneumoniae* is a rare but possible causative agent associated with pyogenic spondylitis.

## Background

Recent years have seen a notable increase in cases of pyogenic spondylitis, especially in elderly populations [1–3]. This rise is attributable to various risk factors prevalent in this group, such as an increased frequency of immunodeficiency disorders and chronic diseases, and more frequent use of invasive spinal procedures [1–3]. The increased use of advanced diagnostic tools such as magnetic resonance imaging or bone scans have also contributed to improved early detection and diagnosis of the disease [2]. Pyogenic spondylitis is associated with a significant mortality rate and frequent relapse [4]. Patients with pyogenic spondylitis also suffer from residual disability, severe functional sequelae, and long-term antibiotic treatment [4].

The most common organism responsible for pyogenic spondylitis infections is *Staphylococcus aureus* [5]. The proportion of gram-negative organisms is 10–30%, compared to 60-80% of gram-positive bacteria [6–10]. Among the gram-negative bacteria, *Escherichia coli* is the most common, followed by *Klebsiella pneumoniae*, which accounts for 1–5% of all infections [6–10]. Intra-abdominal and urinary tract infections (UTIs) are the most common source of pyogenic spondylitis caused by gram-negative bacteria [8, 11].

*K. pneumoniae* present in a healthcare setting, known as “classic *K. pneumoniae*” (cKP), has been reported to be a main causative agent of pneumonia, UTI, and bloodstream infections in immunocompromised patients [12]. “Hypervirulent *K. pneumoniae*” (hvKP), in contrast, tends to be contracted in the community, cause more invasive or metastatic infections, including liver abscess, endophthalmitis, septic lung, osteomyelitis, pyomyositis, and necrotizing fasciitis, and is responsible for more of the infections observed in relatively healthy individuals [12]. To date, 79 capsular types have been identified and associated with various *Klebsiella* species, with the K1 and K2 serotypes known to be associated with hypervirulent features [13, 14]. One recent study showed that virulence genes, such as *iroB*, *iucA*, peg-344, *rmpA*, and *rmpA2*, closely correlate with or are determinant factors of hvKP [15].

*K. pneumoniae* is well-known for its role in pneumonia and other HCA infections [16], but comparatively less information is available concerning pyogenic spondylitis caused by *K. pneumoniae* of the K1 and K2 serotypes or possessing virulent genes. Therefore, we examined the clinical implications of pyogenic spondylitis caused by *K. pneumoniae*, as well as its microbiological features such as the K1 and K2 serotypes, and the virulence genes *iutA*, *rmpA*, *ybtS*, *entB*, *kfu*, *mrkD*, and *allS*.

## Methods

### Study setting and data collection

We conducted a retrospective study at a 1,200-bed tertiary hospital at Jeonbuk National University Hospital in Jeonju, Korea. We reviewed all medical records of patients aged ≥ 19 years who were diagnosed with pyogenic spondylitis, and identified all cases in which *K. pneumoniae* was isolated from blood cultures, tissue cultures, or pus cultures between January 2014 and December 2019. The data collected from each subject included demographic characteristics, clinical signs and symptoms, underlying comorbidities, microbiological data, radiographic findings, laboratory data, surgical treatment, and clinical outcomes. Diagnosis of pyogenic spondylitis was made according to clinical, radiological, and microbiological criteria [17].

### Definition

Healthcare-associated infection (HCA) is defined as a *K. pneumoniae* infection that develops within 48 h of hospital admission in patients (a) hospitalized for two or more days in the preceding 90 days, (b) that received intravenous medication or home wound care in the previous 30 days, (c) that received of hemodialysis, or (d) that reside in a nursing home or long-term care facility [18]. A community-acquired infection is one that occurs in patients with no risk factors for HCA infection for whom the first positive culture for *K. pneumoniae* was identified within 48 h of admission [18]. A hospital-acquired infection is one that is diagnosed through a positive culture for *K. pneumoniae* over 48 h after admission [18].

The neurologic staging is defined as follows: stage 0, no pain; stage 1, back pain at the level of the involved spine; stage 2, radiating pain from the involved spine; stage 3, motor weakness, sensory deficit, and bladder and bowel dysfunction; and stage 4, paralysis [19]. Sequelae are defined as persisting pyogenic spondylitis-associated signs and symptoms that last at least 12 months after the completion of treatment [11].

### Microbiological evaluation and antimicrobial susceptibility

All laboratory tests were performed consistent with routine clinical practice. For cultures, all samples were inoculated onto blood agar and MacConkey agar plates in a 35 °C incubator for 16 to 24 h. The BacT/Alert 3D system (bioMérieux, Durham, NC, USA) was used for blood cultures. The isolated bacteria were identified using a VitekMS system (BioMérieux, Hazelwood, MI, USA) and antimicrobial susceptibility tests were conducted using Vitek 2 AST 211 cards (BioMérieux, Marcy-l’Étoile, France) and interpreted using the VITEK 2 identification system [20]. Multiplex polymerase chain reaction was used to detect K1 and K2 capsular serotypes and virulence genes. Primer sets for *magA* (*wzy*-like polymerase specific to K1 capsular serotype), *wzi* (the gene specifying the K2 capsular serotype), and other virulence genes (*ybtS*, *entB*, *allS*, *kfu*, *iutA*, *mrkD*, and *rmpA*) have been described previously [21]. hvKP is considered detected when both *rmpA* and *iutA* genes are positive [13].

## Results

Over the course of the study a total of six patients with pyogenic spondylitis caused by *K. pneumoniae* were identified; five males and one female (Table 1). The six patients ranged in age from 60 to 84 years. Among the underlying diseases, diabetes mellitus (DM) was confirmed in two of six patients while colon cancer was confirmed in one patient. All patients had community-acquired infections. The sources of infection included a UTI in one patient and a liver abscess in another. All patients had a neurologic staging of 1 or 2. The thoracic spine was involved in two patients while the lumbosacral spine was involved in four cases. Bacteremia was also detected in four patients. The intravenous administration of antimicrobial agents such as ceftriaxone, cefotaxime, ceftazidime, ciprofloxacin, and meropenem continued for between 2 and 7 weeks. After intravenous antibiotic treatment, oral fluoroquinolone was prescribed. No patient underwent decompressive operation or surgical debridement as a result of neurologic complications during antibiotic treatment. Sequalae were observed in two cases but there was no recurrence. Among the six cases, the K1 and K2 serotypes were observed in four cases while hypervirulent strains, which have *rmpA* and *iutA*, were found in five patients (Table 2). One patient died owing to aggravation of septic lung, he did not, however, have the hypervirulent strain or the K1 or K2 serotype. Antimicrobial susceptibility tests showed that all isolates were sensitive to all tested antibiotics (amikacin, ampicillin-sulbactam, aztreonam, cefazolin, cefepime, cefotaxime, cefoxitin, ceftazidime, ertapenem, gentamicin, levofloxacin, meropenem, piperacillin-tazobactam, and tigecycline).

**Table 1:** The clinical features of patients with pyogenic spondylitis caused by *Klebsiella pneumoniae*

	**Case 1**	**Case 2**	**Case 3**	**Case 4**	**Case 5**	**Case 6**
Age	67	60	52	84	61	78
Sex	M	M	F	M	M	M
Underlying disease	Colon cancer	DM, hypertension	No	No	Liver cirrhosis, CKD	DM
Type of infection	CAI	CAI	CAI	CAI	CAI	CAI
Spinal procedure						
Vertebroplasty	Not done	Not done	Not done	Not done	Not done	Not done
Prosthesis	Not done	Not done	Not done	Not done	Not done	Not done
Source of infection	No	No	No	UTI	No	Liver abscess
Neurologic staging	Stage 1	Stage 1	Stage 1	Stage 2	Stage 2	Stage 2
Radiologic finding						
Involved area	T-spine	T-spine	L-S spine	L-S spine	L-S spine	L-S spine
Involved number	1 to 2 VB	1 to 2 VB	1 to 2 VB	1 to 2 VB	1 to 2 VB	> 5 VB
Combined abscess	Epidural abscess	No	No	Epidural, psoas abscess	Epidural, psoas abscess	Epidural, psoas abscess
Laboratory finding						
WBC(10^3^/µL)	15.00	9.37	18.57	22.15	8.03	7.97
CRP(mg/L)	22	129	362	204	215	281
Procalcitonin(ng/mL)	Not checked	0.26	12.41	0.85	74.76	62.78
Bacteremia	No	No	Yes	Yes	Yes	Yes
Tissue culture and sensitivity	Yes	Yes	No	Yes	No	No
Symptoms and signs						
Fever	No	Yes	Yes	Yes	Yes	Yes
Back pain	Yes	Yes	Yes	Yes	Yes	Yes
Metastatic infection	No	No	Pneumonia	Pneumonia	No	No
Outcome						
30-day mortality	Alive	Alive	Alive	Dead	Alive	Alive
Sequalae	No	Yes	No	N.A	Yes	Yes
Recur	No	No	No	N.A	No	No
Treatment						
Antibiotics	CAZ IV	CRO IV, LVX PO	CRO IV, CIP PO	VAN IV, MEM IV	CTX IV, CIP PO	CRO IV, CIP PO
Duration	6 weeks	4 weeks, 3 months	7 weeks, 2 months	2 weeks	2 weeks, 7 months	7 weeks, 3 months
Operation	Not done	Not done	Not done	Not done	Not done	Not done

CAI, community acquired infection; CKD, chronic kidney disease; DM, diabetes mellitus; UTI, urinary tract infection; VB, vertebral body; WBC, white blood cells; CRP, C-reactive protein; CAZ, ceftazidime; CRO, ceftriaxone; LVX, levofloxacin; CIP, ciprofloxacin; VAN, vancomycin; MEM, meropenem; CTX, cefotaxime; IV, intravenous, PO; oral

**Table 2:** The serotypes and virulent factors of *Klebsiella pneumoniae*

	**Case 1**	**Case 2**	**Case 3**	**Case 4**	**Case 5**	**Case 6**
**Serotype**	**Non K1/K2**	**K1**	**K1**	**Non K1/K2**	**K2**	**K1**
Virulent factor						
*rmpA*	Positive	Positive	Positive	Negative	Positive	Positive
*iutA*	Positive	Positive	Positive	Negative	Negative	Positive
*allS*	Negative	Positive	Positive	Positive	Positive	Positive
*entB*	Positive	Positive	Positive	Positive	Negative	Positive
*kfu*	Negative	Positive	Positive	Positive	Negative	Positive
*mrkD*	Positive	Positive	Positive	Positive	Positive	Positive
*ybtS*	Positive	Positive	Positive	Negative	Negative	Positive

## Discussion

This study suggests that *K. pneumoniae* is a clinically rare but significant pathogen of pyogenic spondylitis. To our knowledge, this is the first study to investigate K1 and K2 serotypes and virulence factors of pyogenic spondylitis-causing *K. pneumoniae*. Of the six strains tested, four were K1 and K2 serotypes while five were hypervirulent strains, suggesting that most *K. pneumoniae* that causes pyogenic spondylitis is hvKP.

Consistent with other studies concluding that intra-abdominal infections and UTI predisposing factors for pyogenic spondylitis caused by gram-negative bacteria, the UTI in Case 4 and liver abscess in Case 6 were identified as presumed sources of pyogenic spondylitis [8, 11]. The primary location of vertebral involvement, the number of invaded vertebral bodies, and the rate of positive blood culture observed in these patients were consistent with previous studies [8, 11]. Although there is no officially-recognized recommended treatment period, 8 weeks has been suggested as appropriate to prevent recurrence [11]. In this study, the intravenous antibiotics used included ceftriaxone, cefotaxime, ceftazidime, ciprofloxacin, and meropenem. The period of intravenous antibiotic administration of was 2–7 weeks, after which oral fluoroquinolone was maintained. In no case was recurrence observed after the conclusion of treatment. Because fluoroquinolone has a high oral bioavailability and good penetration into bone tissue, oral fluoroquinolone may be a good therapeutic option for prolonged antibiotic therapy [11]. For infectious spondylitis caused by gram-negative bacteria, 49.2% of patients undergo surgical treatment, but in this study none of the patients underwent surgery during antibiotic treatment [11]. This antibiotics used in Park et al.‘s study were fluoroquinolone and third-generation cephalosporin, which are similar to the antibiotics used in our study [11].

No standard definition of hvKP has yet been established. Hypervirulence in *K. pneumoniae* can be defined as the ability of the organism to cause invasive infections in healthy adults through metastatic dissemination from a primary infection site [22]. Diverse virulence factors are associated with a hypervirulent phenotype [12], including the production of hypercapsule, lipopolysaccharide, siderophores, and fimbriae (pili) [12]. Hypervirulence as a phenomenon is not thought to be associated with a single gene, but rather appears to be the result of complex interactions between multiple genetic determinants [13, 22]. The aerobactin gene cluster *iucABCD*, aerobactin transporter *iutA* (biosynthetic genes for the siderophore aerobactin), and *rmpA* or *rmpA2* (which trigger hypercapsule production) have been regarded as good diagnostic candidates for hypervirulence [13]. In our study, five of the six strains were hypervirulent based on this definition, and in each case the strain was the causative pathogen of the infectious spondylitis. Although we were limited by the small number of samples available for testing, our findings do suggest that the hypervirulent strain may be more associated with pyogenic spondylitis than the classic strain. A study that compares the frequency of hvKP and cKP-induced pyogenic spondylitis with a greater number of study subjects is needed to confirm this hypothesis.

*K. pneumoniae* is a well-known multidrug-resistant bacteria with emerging carbapenemase-producing strains. In contrast, hvKP remains susceptible to most clinically used antibiotics [12]. All six patients studied showed community-acquired infection, and each of the identified strains proved to be susceptible to all antimicrobial agents studied. However, the emergence of hvKP-producing extended spectrum beta-lactamase (ESBL) or carbapenemase has been reported [23, 24]. As a pyogenic spondylitis treatment, the administration of intravenous antibiotics or highly bioavailable oral agents is recommended to continue for longer than 6 weeks [25]. If hvKP strains producing ESBL or carbapenemase cause pyogenic spondylitis, the selection of antimicrobial agents available in these cases is very limited, and it is unlikely that those available would exert a sufficient therapeutic effect. Moreover, the treatment of multiple sites infected by hvKP, including metastatic infections, is a therapeutic challenge owing to the poor penetration of certain antimicrobial agents when used to treat pyogenic spondylitis [13]. Although ESBL or multidrug-resistant hvKP were reported neither in this study nor previous studies concerning pyogenic spondylitis, continuous surveillance is required.

This study had some limitations. Salmochelin (*iroA* gene cluster, *iroBCDE*) and peg-344 were recently reported to be significant virulent factors specific to hvKP, though these were not tested in this study [15] nor did we test for the presence of *rmpA2*. Another limitation was the small number of cases, though we note that all obtainable cases over a six-year period were included. Finally, we investigated only some virulence factors, and declined to explore any virulence factors through an animal study. While hypervirulence could be demonstrated easily in a mouse infection model or *Galleria mellonella* infection model, we were unable to perform these tests. We defined microbial hypervirulence exclusively by reference to the presence of virulence genes [26].

## Conclusion

This study suggests that hvKP is a rare but possible causative agent associated with pyogenic spondylitis. Although *K. pneumoniae* infection constitutes a relatively minor proportion of all cases, its clinical importance should be evaluated through larger studies, as treatment can become more complicated when resistance to antimicrobial agents increases.

## Data Availability

The data that support the findings of this study ara available from the corresponding author upon reasonable request.

## Abbreviations

cKP: classic *K. pneumonia*
hvKP: hypervirulent *K. pneumonia*
HCA: healthcare-associated infection
UTI: urinary tract infection
ESBL: extended spectrum β lactamase
